# The Mayo Brothers: Pioneers of Modern Medicine and Patient-Centered Care

**DOI:** 10.7759/cureus.71269

**Published:** 2024-10-11

**Authors:** Karthik Gnanapandithan, Ricardo J Pagán, Jennifer Cowart

**Affiliations:** 1 Hospital Medicine, Mayo Clinic, Jacksonville, USA

**Keywords:** charles mayo, historical vignette, mayo brothers, mayo clinic, patient-centered care, william mayo

## Abstract

The Mayo brothers, Drs. William James Mayo and Charles Horace Mayo, are celebrated for their groundbreaking contributions to modern medicine. This review examines their journey from humble beginnings to establishing the Mayo Clinic, a world-renowned medical institution. They pioneered a collaborative, team-based approach to medicine, which was revolutionary then. They emphasized the importance of specialization and interdisciplinary cooperation, laying the foundation for modern healthcare practices like integrated care and the rise of group medical practices. Their impact continues through the Mayo Clinic's patient-centered approach, which has influenced countless healthcare institutions worldwide.

## Introduction and background

The evolution of modern medicine has been significantly shaped by visionary figures whose contributions continue to influence current practices. Among these figures, the Mayo brothers stand out for their pioneering work in clinical practice, medical education, and research [1]. Dr. William James Mayo, Dr. Charles Horace Mayo, and their father, Dr. William Worrall Mayo, laid the foundations for the Mayo Clinic in 1889, which has now become synonymous with excellence in healthcare [2]. This review explores their remarkable journey, contributions to the medical field, and enduring legacy. We also discuss how their innovative approach to patient-centered medicine, integrated clinical practice, education, and research has left an indelible mark on the medical field, influencing practices and healthcare systems globally.

## Review

Early life and education

**Figure 1:**
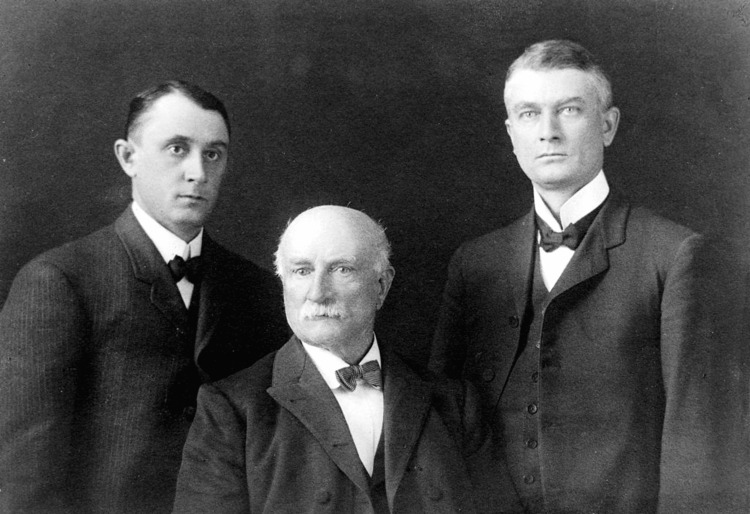
Portrait of William Worrall Mayo with his sons, Charles Horace Mayo (left) and William James Mayo (right) Image Credit: Wellcome Library, London via Wikimedia Creative Commons [2]

The roots of the Mayo Clinic trace back to Dr. William Worrall Mayo [1], a British immigrant who settled in Rochester, Minnesota, in 1863. He was a general practitioner who also served as an examining physician for the US Army recruitment board. His medical practice and principles laid the foundation for the Mayo Clinic and the Mayo way of delivering care. William James Mayo ("Will"), born in 1861, and Charles Horace Mayo ("Charlie"), born in 1865, were heavily influenced by their father's dedication to medicine (Figure 1) [2]. Starting at an early age, the Mayo brothers assisted their father's medical practice, compounding pills and solutions prescribed to patients and performing other basic patient care tasks. These early days played a critical role in shaping their approach to medicine. Growing up in a household deeply engaged with medical practice with their father's emphasis on practical medicine, they were exposed to hands-on learning from a young age. This, combined with formal medical education at prestigious institutions like the University of Michigan (Will) and the Chicago Medical College (Charlie), laid the foundation for their later innovations [2]. These formative experiences fostered their understanding of the importance of surgical precision and patient-centered care, which became hallmarks of their approach to surgery and the development of integrated medical care.

Founding of the Mayo Clinic

On August 21, 1883, Rochester, Minnesota, was impacted by a tornado, leaving widespread destruction in a major part of the city. Temporary facilities staffed by physicians and other medical staff cared for the wounded. The Sisters of St. Francis, whose primary role was in school education, rose to the occasion and volunteered their help in the event of the disaster. Mother Alfred Moes partnered with Dr. William Worrall Mayo and his sons. The sisters proposed setting up a medical facility, managing it, and training future nurses with the Mayo family in charge of patient care [2].

**Figure 2:**
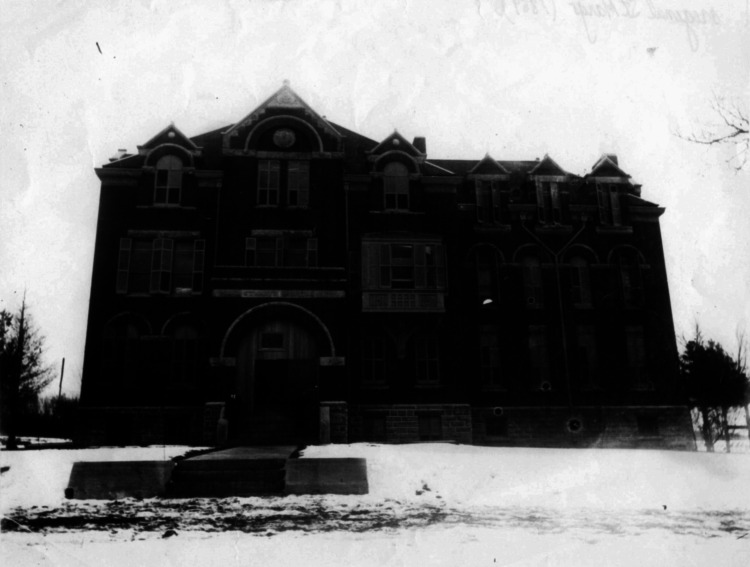
Saint Mary's Hospital, Rochester, Minnesota, 1889 Image Credit: [3]; published with permission

Saint Mary's Hospital was founded in 1889 (Figure 2) [3] and marked the beginning of what would evolve into the Mayo Clinic [4]. As a collaborative effort between the Mayo family and the Sisters of St. Francis, the hospital was created in the aftermath of a natural disaster but driven by a vision to provide continued high-quality medical care. This collaboration between physicians and the religious community set the stage for developing a unique, team-based approach to healthcare. Patient care was prioritized, and physicians, nurses, and administrators worked together toward a common goal. This cooperative spirit would later become a defining feature of the Mayo Clinic's structure, fostering an environment where innovation, teamwork, and patient-centered care thrived. The success of Saint Mary's Hospital laid the groundwork for the Mayo Clinic's eventual transformation into a world-renowned medical institution [4].

Innovations and contributions

The Mayo Clinic's success can be attributed to the brothers' relentless pursuit of innovation. Will and Charlie Mayo were talented surgeons who also traveled to learn new techniques and protocols from other surgical practices to improve patient care [5]. They visited the Johns Hopkins Hospital and trained under renowned surgeon William Halsted, where they were introduced to the antiseptic techniques developed by Joseph Lister [6], which they brought back to Rochester. From its inception, Saint Mary's Hospital was one of the first centers worldwide to employ the new principles of disinfection and antiseptic practices. By meticulously preventing infections, they improved surgical outcomes and reduced mortality.

Will Mayo specialized in abdominal and pelvic surgeries, while Charlie Mayo leaned toward head, neck, and neurological surgeries. Their meticulous approach to surgical procedures and emphasis on sterile techniques and postoperative care set new benchmarks in the field. The Mayo brothers introduced pioneering surgical methods that significantly improved patient outcomes [5]. They also introduced novel methods in thyroid surgery and developed pioneering approaches to gallbladder and gastric surgeries [7]. They were also early adopters of X-ray technology, recognizing its potential in diagnostics and treatment planning [8]. The collaborative, multidisciplinary nature of the Mayo Clinic allowed for the continuous refinement of these techniques, with surgeons sharing knowledge in real time, something rare for the era. As their popularity surged due to the superior results of their surgeries at Saint Mary's Hospital, more patients came to them. The referring physicians used the term "The Mayo Clinic," but the name was not formalized until 1914 [2]. The medical community highly regarded their work, receiving praise at national and international surgical conferences. These innovations not only advanced the field of surgery but also positioned the Mayo Clinic as a leader in medical education and research.

Impact on medical education

The Mayo brothers' contributions to medical education were transformative. They recognized the need for structured training programs to produce competent, skilled physicians. In the words of Dr. Charles Mayo, "There are two objects of medical education: to heal the sick and to advance the science" [9]. One of their most lasting contributions was their belief in the value of hands-on training under the supervision of experienced physicians, an approach that became the cornerstone of the residency system [10]. This model became the gold standard for medical training, influencing institutions worldwide. The three-year physician training programs were started in 1915 in partnership with the University of Minnesota [11]. The first Ph.D. in biochemistry was conferred in 1917. While the degrees in earlier years were granted with the University of Minnesota affiliation, the Mayo Clinic began to award master's and Ph.D. degrees independently in 1989 [11].

The Mayo Clinic became a hub for medical education, attracting physicians from around the world, including Europe and Asia. The brothers emphasized the importance of continuous learning and professional development, establishing residency programs, and fostering an environment of academic excellence. They developed structured educational programs that combined clinical practice with research, allowing physicians to continually refine their skills while staying current with medical advancements. This model directly influenced the development of modern postgraduate medical education, with many of today's residency programs mirroring the comprehensive, interdisciplinary approach the Mayo brothers pioneered. Their commitment to lifelong learning persists in today's continuing medical education requirements, ensuring that physicians stay current on the latest practices. The Mayo Clinic remains a leader in medical education, with its residency and fellowship programs setting the standards for medical education globally. Their commitment to education extended to publishing extensively in medical journals, contributing to the body of medical knowledge, and setting standards for clinical practice. Their emphasis on continuous education ensured that physicians remained abreast of the latest medical advancements. They organized conferences, lectures, and workshops, creating a vibrant academic environment encouraging knowledge sharing and professional growth [12].

Research contributions

The Mayo brothers were pioneers in promoting medical research. They understood that advancements in medical science were crucial for improving patient care. They established dedicated research laboratories and encouraged physicians to engage in research projects [10]. Their research into the thyroid disorders helped the Mayo Clinic become a global leader in thyroid surgery and treatment of goiter in the 1900s [5]. The Mayo Clinic was an early adopter of medical records research. Dr. Henry Plummer, who joined the Mayo Clinic in 1901, designed one of the first integrated medical record systems [13]. This system allowed for the systematic study of patient outcomes, a precursor to modern-day clinical research and evidence-based medicine. These early achievements set the stage for the Mayo Clinic's continuous contributions to advancing healthcare through research, education, and innovation. Their collaborative research led to numerous breakthroughs and innovations, even after their deaths. One of the best examples is the receipt of the Nobel Prize for Medicine in 1950 by two Mayo Clinic researchers, Dr. Edward C. Kendall (Mayo's first rheumatologist) and Dr. Philip S. Hench (researcher in biochemistry), for the identification of cortisone [14]. The Mayo Clinic's research contributions are vast and continue to serve patients worldwide. The institution has made significant strides in medical science, from developing new surgical techniques to pioneering treatments for various diseases. The Mayo brothers' commitment to research ensured that the clinic remained at the cutting edge of medical innovation.

Military service

Will Mayo served as a colonel for the US Army Medical Corps in World War I and was a chief advisor in the Surgeon General's office [15]. Charlie Mayo was a colonel involved in the surgical care of the soldiers as an associate chief advisor. Later, they both served in the National Defense Council's General Medical Board. The Mayo Clinic played a significant role in the background of World War I [16]. Will and Charlie organized educational camps to train military doctors in the latest medical and surgical techniques. Despite their commitments to the Army, they ensured that one of them was always available at the Mayo Clinic for its patients. After the war ended in November 1918, both Mayo brothers were promoted in the US Army Reserve to the rank of brigadier general. They also received the United States Distinguished Service Medal as an appreciation for their service to the country during the crucial wartime [17]. Their wartime experiences dealing with large-scale injuries and complex surgeries directly influenced their post-war medical practice, driving further innovation in trauma care. The Mayo Clinic's Military Medicine Program [18] was developed to better understand and address the unique needs of service members on and off the battlefield. It serves as a center stage for Mayo Clinic researchers and potential collaborators interested in military and defense-related research initiatives.

Notable achievements and legacy

**Figure 3:**
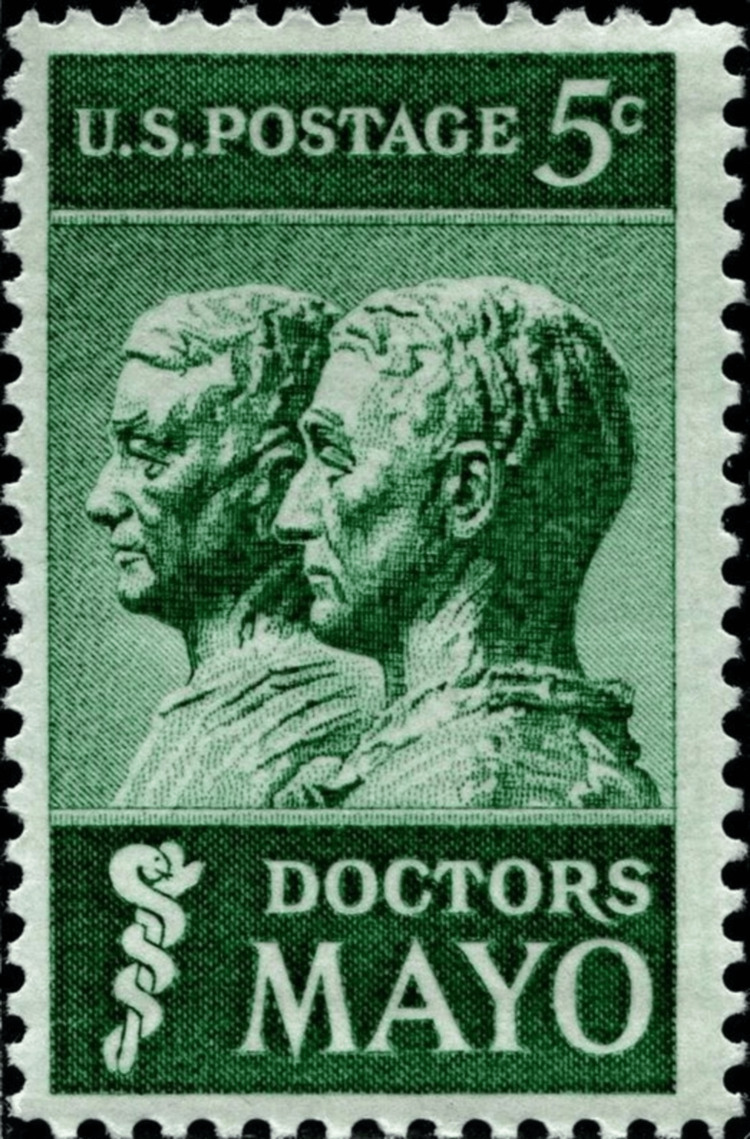
Postage stamp issued in 1964 by the United States Postal Service in honor of the Mayo brothers Image Credit: [15]; published with permission

The Mayo brothers' vision extended beyond clinical practice. In 1915, they established the Mayo Foundation for Medical Education and Research, ensuring their mission of integrating practice, education, and research would continue. The brothers donated their lifetime savings to the Foundation [11]. All earnings from the clinical practice were funneled to a trust to support stipends for residents and fellows training at the Mayo Clinic. The foundation supported numerous research initiatives, leading to significant advancements in various medical fields [10]. Will and Charlie Mayo were honored by medical societies the world over. They presided over the American College of Surgeons and served in lead positions in almost every major medical society during their lifetime [1]. In 1964, the United States Postal Service issued a stamp to honor the Mayo brothers (Figure 3) [19].

Under their leadership, the Mayo Clinic achieved numerous milestones. They developed the integrated group practice model, wherein specialists from various fields collaborated to provide comprehensive care [20]. This model revolutionized medical practice, ensuring patients received well-rounded and coordinated treatment. This approach not only improved patient care but also fostered innovation and discovery. The Mayo Clinic continues to be well known for its multidisciplinary approach to managing complex patients [21].

The journey to establishing the Mayo Clinic was not without challenges [22]. Financial constraints and skepticism from the medical community marked the early years. However, the Mayo brothers' determination and innovative spirit helped them overcome these obstacles. They leveraged collaboration and teamwork to build a strong and resilient institution. Their ability to attract talented physicians and foster a culture of mutual respect and support was vital to their success. The Mayo Clinic's growth from a small hospital to a world-renowned medical center is a testament to their vision and leadership.

**Figure 4:**
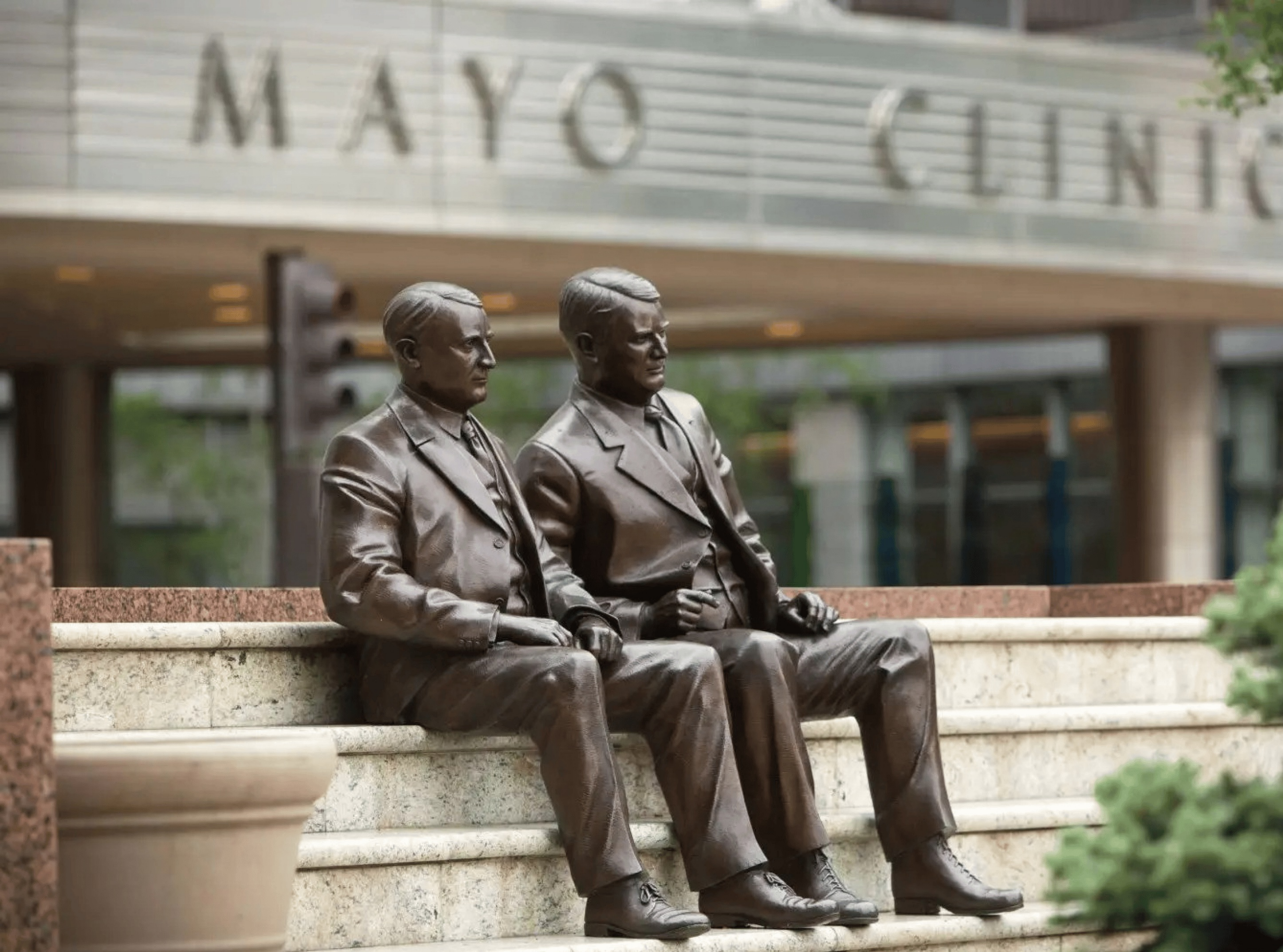
The My Brother and I monument, a bronze sculpture of the Mayo brothers outside the Mayo Clinic in Rochester, Minnesota Image Credit: [20]; published with permission

Will and Charlie Mayo died in 1939 from unrelated medical illnesses [2]. While it was speculated then that the Mayo Clinic would not survive without them, history has proven the critics wrong. The Mayo brothers left a rich legacy, which is evident in the continued success and global reputation of the Mayo Clinic. Seventy years after their passing, the brothers were inducted into the Healthcare Hall of Fame in 2009 [23]. The institution remains at the forefront of medical research, education, and patient care, embodying the principles and values instilled by the Mayo brothers. The My Brother and I monument, a bronze sculpture of the Mayo brothers resting on steps outside the Mayo Clinic in Rochester, Minnesota, was installed in 2002 (Figure 4) [24].

Mayo Clinic values and vision

A values-based organization, the Mayo Clinic's values are derived from the lives and practices of the Mayo brothers and the Sisters of St. Francis. Quotes and sayings from the Mayo brothers have been incorporated into Mayo Clinic values and culture. A 1910 commencement address by Dr. Will included this statement [25]: "The best interest of the patient is the only interest to be considered, and in order that the sick may have the benefit of advancing knowledge, union of forces is necessary." This became the Mayo Clinic's principal value: "The needs of the patient come first." In addition, the Mayo Clinic created the acronym RICH TIES (Respect, Integrity, Compassion, Healing, Teamwork, Innovation, Excellence, Stewardship), commonly used among the staff to recollect the fundamental values easily [26].

A commitment to patient-centered care was at the core of the Mayo brothers' philosophy. They firmly believed the patient's needs should always come first [27]. This principle guided every aspect of their practice, from clinical care to research and education. Their vision for medicine extended beyond individual patient care. They advocated for ethical standards and professional conduct, emphasizing the importance of compassion, integrity, and excellence in medical practice [27]. Their holistic approach to healthcare, which considered the physical, emotional, and social aspects of health, was revolutionary at the time and continues to influence modern medical practice. Medical institutions worldwide have adopted the Mayo Clinic's model of integrated clinical practice, education, and research [9]. The clinic's emphasis on multidisciplinary care and continuous improvement has set a global standard for healthcare delivery. The principles and practices of the Mayo brothers continue to remain the backbone of the ongoing work of the Mayo Clinic.

Future directions

The Mayo Clinic continues to evolve as a leader in healthcare, research, and education, focusing on integrating emerging technologies such as artificial intelligence, precision medicine, telehealth, and advanced diagnostics. The clinic's "Cure. Connect. And Transform" strategy [28] outlines their commitment to harnessing innovation to meet the challenges of future healthcare delivery. The Mayo Clinic in Rochester, Minnesota, has consistently ranked first in the "US News Best Hospitals" rankings [29], a testament to its unparalleled excellence in patient care, research, and education. Additionally, the Mayo Clinic's campuses in Arizona and Florida have been ranked as the best hospitals in their respective states [29], further demonstrating the institution's national leadership in healthcare across multiple locations. Per their 2022 factsheet, the Mayo Clinic employs over 76,000 staff members, including more than 5,500 physicians and scientists, and trains thousands of medical residents and fellows across various specialties. In 2023 alone, the clinic cared for about 1.3 million patients from over 130 countries. The institution played a significant role in combating the COVID-19 pandemic. Mayo Clinic scientists were instrumental in developing convalescent plasma therapies and contributed to vaccine research, sharing critical public health data to guide national responses [30,31]. This ongoing commitment to innovation and collaboration ensures that the Mayo Clinic will remain a key player in addressing future healthcare challenges.

## Conclusions

The Mayo brothers' contributions to medicine are immeasurable. Their innovative approach to clinical practice, medical education, and research has left a legacy shaping modern medicine. The principles they championed, namely, patient-centered care, teamwork, continuous learning, and ethical conduct, remain at the core of the Mayo Clinic's mission. As we reflect on their journey, it is clear the Mayo brothers were pioneers of their time and visionaries whose impact will continue to be felt for generations. As healthcare evolves, the Mayo Clinic remains committed to leading the way in innovation, patient-centered care, and medical research. By staying true to the collaborative and patient-first ethos established by the Mayo brothers, the institution will continue to advance personalized care, offering solutions for the complex medical challenges of tomorrow while maintaining its legacy of compassion and excellence.
